# bcl-2 gene enables rescue from in vitro myelosuppression (bone marrow cell death) induced by chemotherapy.

**DOI:** 10.1038/bjc.1994.321

**Published:** 1994-09

**Authors:** S. Kondo, D. Yin, J. Takeuchi, T. Morimura, Y. Oda, H. Kikuchi

**Affiliations:** Department of Neurosurgery, National Utano Hospital, Kyoto, Japan.

## Abstract

**Images:**


Br. J. Cancer (1994), 70, 421-426 © Macmillan Press Ltd., 1994

### *bcl*-2 gene enables rescue from *in vitro* myelosuppression (bone marrow cell death) induced by chemotherapy

S. Kondo[1], D. Yin[1], J. Takeuchi[1], T. Morimura[1], Y. Oda[2] & H. Kikuchi[2]

[1]*Department of Neurosurgery, National Utano Hospital, Kyoto 616, Japan;* [2]*Department of Neurosurgery, Faculty of Medicine, Kyoto University, Kyoto 606, Japan.*

**Summary** Recent studies have shown that the use of cytokines such as granulocyte colony-stimulating factor (G-CSF) to ameliorate chemotherapy-induced myelosuppression may enhance the viability of tumour cells with functional receptors for these cytokines. In this study, therefore, we used murine bone marrow (BM) cells in an *in vitro* model in an attempt to determine whether topoisomerase inhibitors (camptothecin, etoposide and doxorubicin) induce myelosuppression (BM cell death) and whether novel treatments other than the administration of G-CSF can be used for rescue from myelosuppression. DNA fragmentation assay, ultrastructural analysis and cell cycle analysis demonstrated that these chemotherapeutic agents induced apoptosis in BM cells. We demonstrated in addition that enforced expression of the *bcl*-2 gene in BM cells by MPZenNeo (*bcl*-2) retroviral gene transfer increased resistance to the apoptosis induced by these agents. These findings suggest the possibility that enforced expression of the *bcl*-2 gene in BM cells using gene transfer techniques may enable rescue from chemotherapy-induced myelosuppression.

The intensive use of chemotherapeutic agents is a mainstay in the current treatment of malignant diseases. However, myelosuppression is a common and serious complication of treatment with these agents since most lack specificity for malignant cells. Therefore, reduction in the degree of duration of chemotherapy-induced myelosuppression may decrease the morbidity of chemotherapy and enhance tumour control.

In general, rhG-CSF[4] is well tolerated within a range of doses effective in increasing the rate of myeloid recovery after high-dose chemotherapy (Antman *et al.*, 1988; Brandt *et al.*, 1988). However, Lotem and Sachs (1992) have recently suggested that the use of cytokines such as G-CSF to ameliorate myelosuppression that follows the use of chemotherapeutic agents or radiation in cancer therapy may not only stimulate the recovery of normal hematopoietic cells but also enhance the viability of leukaemic cells or non-haematopoietic tumour cells with functional receptors for these cytokines. Therefore, in the case of tumours with G-CSF receptors, the application of novel treatments other than the administration of rhGM-CSF will be necessary for amelioration of the myelosuppression induced by chemotherapy.

While much information is available on the cellular targets and mechanisms of action of chemotherapeutic agents, little is known about how they actually induce cell death. Recent studies have shown that the most likely mechanism of cell death following exposure to chemotherapeutic agents is apoptosis (programmed cell death) (Eastman, 1990; Martin *et al.*, 1990; Sorenson *et al.*, 1990; Martin & Cotter, 1991; Walker *et al.*, 1991; Onishi *et al.*, 1993). Therefore, apoptosis of bone marrow (BM) cells may be the main mechanism of myelosuppression.

Drugs that interact with DNA topoisomerase, such as CPT, VP-16 and doxorubicin, have been found to be particularly useful in chemotherapeutic treatment, but do tend to induce myelosuppression (Glisson & Ross, 1987; Kohn *et al.*, 1987; Zwelling, 1989). Recently, CPT and VP-16 have been shown to induce apoptosis with DNA cleavage in thymocytes (Walker *et al.*, 1991; Onishi *et al.*, 1993) and in concanavalin A-stimulated splenocytes (Jaxel *et al.*, 1988). In addition, doxorubicin is known to induce apoptosis in the murine small intestinal tract (Thakkar & Potten, 1992) and in thymocytes (Onishi *et al.*, 1993). In this study, therefore, we used DNA fragmentation assay, ultrastructual analysis and cell cycle analysis in attempting to determine whether CPT, VP-16 and doxorubicin induce apoptosis in murine BM cells.

On the other hand, forced expression of *bcl*-2, a gene implicated in the genesis of follicular lymphoma (Tsujimoto *et al.*, 1984; Bakhshi *et al.*, 1985; Cleary & Sklar, 1986), has been shown to prevent apoptosis of factor-dependent myeloid cells and pro-B-cell lines cultured in the absence of growth factor (Cleary & Sklar, 1985; Vaux *et al.*, 1988). In addition, expression of *bcl*-2 has been shown to increase the resistance of cells to ethanol, methotrexate and heat shock (Nunez *et al.*, 1990). We therefore wished to determine whether the *bcl*-2 gene could prevent BM cell death induced by chemotherapeutic agents. Accordingly, we introduced a human *bcl*-2 gene into BM cells by MPZenNeo (*bcl*-2) retroviral gene transfer (Cleary & Sklar, 1985; Tsujimoto, 1989).

In this study, we also attempted to determine whether a high rate of expression of the *bcl*-2 gene in BM cells can prevent cell death induced by chemotherapy.

### Materials and methods

### *Bone marrow cells*

Murine bone marrow (BM) cells were obtained from the femurs of 5- to 6-week-old Balb/c female mice. Marrow plugs were flushed out with phosphate-buffered saline (PBS), passaged through a 23 gauge needle, washed and resuspended in complete RPMI-1640 medium supplemented with 10% heat-inactivated fetal calf serum (FCS) (Gibco, Grand Island, NY, USA), 4 mM glutamine, 50 U ml$^{-1}$ penicillin and 50 $\mu$g ml$^{-1}$ streptomycin at a density of $10^6$ cells ml$^{-1}$. Macrophages and other adherent cells were removed after an 18 h period of incubation at 37°C in 5% carbon dioxide in tissue culture flasks as previously described (Strassman *et al.*, 1988). Non-adherent cells were collected and resuspended in complete medium and cultured with 10 ng ml$^{-1}$ rhG-CSF kindly supplied by Chugai Pharmaceuticals (Tokyo, Japan).

### *Chemotherapeutic agents*

CPT was purchased from Sigma (St Louis, MO, USA). VP-16 was a generous gift from Nippon Kayaku (Tokyo, Japan). These agents were obtained in powder form, from which 3.5 mg ml$^{-1}$ or 10 mg ml$^{-1}$ stock solution was prepared in dimethylsulphoxide. Doxorubicin was a generous gift from Kyowa Hakko Kogyo (Tokyo). It was also obtained in powder form, from which a 1.0 mg ml$^{-1}$ stock solution was prepared in normal saline.

Correspondence: S. Kondo, Department of Neurosurgery, National Utano Hospital, 8-Ondoyama-cho, Narutaki, Ukyo-ku, Kyoto 616, Japan.
Received 9 November 1993; and in revised form 29 March 1994.

*Retrovirus infection*

A 942 bp blunted (*Eco*RI/*Taq*I cDNA fragment containing the entire human *bcl*-2 coding sequence (Tsujimoto, 1989) was inserted into the blunted *Xho*I site of the retroviral vector MPZenSVNeo, allowing expression of *bcl*-2 from the MPSV long terminal repeat (Cleary & Sklar, 1985; Hariharan *et al.*, 1988). Murine fibroblast lines secreting MPZenNeo (*bcl*-2) or MPZenNeo virus free of helper virus were obtained by electroporating the ψ2 packaging line (Mann *et al.*, 1983) with retroviral plasmid DNA. BM cells were infected by adding the filtered supernatant of the virus-producing ψ2 fibroblast cells with incubation for 24 h. Infected BM cells were selected in 800 $\mu g\ ml^{-1}$ G418. The production of the *bcl*-2 gene in BM cells was assessed by immunoprecipitation using anti-human Bcl-2 MAb (Biochemicals, Tokyo, Japan).

*Immunoprecipitation*

BM cells were washed with methionine-free medium containing 5% FCS, suspended at $2.5 \times 10^6$ cells $ml^{-1}$ in the same medium supplemented with 250 $\mu Ci\ ml^{-1}$ [$^{35}S$]L-methionine (Amersham, Arlington Heights, IL, USA) and cultivated for 5 h. The cells were harvested, washed with PBS and lysed as previously described (Kondo *et al.*, 1992). The lysate was mixed with anti-human Bcl-2 MAb, and the immune complexes were precipitated with protein A–Sepharose (Amersham) and analysed on a 12% sodium dodecyl sulphate–polyacrylamide gel.

*Cell viability assays*

BM cells were seeded at $10^5$ cells per well (1.0 ml) in 24-well plates and treated with various chemotherapeutic agents at clinically relevant concentrations. Each day an aliquot was examined microscopically using trypan blue to determine percentage cell viability.

*Inhibition of RNA and protein synthesis*

To determine whether inhibition of RNA or protein synthesis results in inhibition of the cytotoxicity induced by chemotherapeutic agents, BM cells were pretreated for 15 min with actinomycin D (80 $ng\ ml^{-1}$) or cycloheximide (0.7 $\mu g\ ml^{-1}$) prior to chemotherapy. Higher concentrations of actinomycin D and cycloheximide caused cytotoxicity in BM cells by themselves. Changes in cytotoxicity were determined by trypan blue exclusion.

*Analysis of DNA fragmentation in agarose gel*

This assay was performed as previously described (Ishida *et al.*, 1992). Briefly, harvested BM cells ($1 \times 10^7$) were centrifuged and washed twice with cold PBS. The cell pellet was lysed in 1.0 ml of a buffer consisting of 10 mM Tris–HCl, 10 mM EDTA and 0.2% Triton X-100 (pH 7.5). After 10 min on ice, the lysate was centrifuged (13,000 *g*) for 10 min at 4°C in an Eppendorf microfuge. Then, the supernatant (containing RNA and fragmented DNA, but not intact chromatin) was extracted first with phenol and then with phenol–chloroform:isoamyl alcohol (24:1). The aqueous phase was made to 300 mM sodium chloride and nucleic acids were precipitated with two volumes of ethanol. The pellet was rinsed with 70% ethanol, air dried and dissolved in 20 μl of 10 mM Tris–HCl–1 mM EDTA (pH 7.5). Following digestion of RNA with RNAse A (0.6 $mg\ ml^{-1}$, at 37°C for 30 min), the sample was electrophoresed in a 2% agarose gel with Boyer's buffer (50 mM Tris–HCl, 20 mM sodium acetate, 2 mM EDTA and 18 mM sodium chloride, pH 8.05). DNA was then visualised with ethidium bromide staining.

*Ultrastructural analysis*

To determine morphologically whether apoptosis is induced in BM cells by topoisomerase inhibitors, parental and *bcl*-2-expressing BM cells treated with 10 $\mu g\ ml^{-1}$ VP-16 for 48 h were examined at the ultrastructural level. Briefly, $2 \times 10^6$ BM cells were harvested, washed in PBS, pelleted, prefixed in 2.0% glutaraldehyde for 2 h and washed in 0.1 M phosphate buffer (pH 7.4), followed by post-fixation with 1.0% osmium tetroxide for 2 h. Samples were embedded in Econ 812, sectioned and stained for 20 min in 2.0% aqueous uranyl acetate and for 2 min in lead citrate. Grids were viewed using a JEM-1200EX electron microscope (NEC, Tokyo, Japan).

*Flow cytometry*

Parental and *bcl*-2-expressing BM cells were treated with 10 $\mu g\ ml^{-1}$ VP-16 for 48 h. Then, $2.0 \times 10^6$ cells were fixed with 2 ml of 70% ethanol on ice for 15 min, pelleted and stained with propidium iodide (50 $\mu g\ ml^{-1}$ in PBS) containing 0.5 $mg\ ml^{-1}$ RNAse A for an additional 30 min on ice, prior to analysis of DNA content by flow cytometry. Cells were tested for cell cycle position using a FACScan flow cytometer (Becton Dickinson, Mountain View, CA, USA) equipped with CellFIT version 2.0 software. The SOBR (sum of broadened rectangles) model provided by this software was used to estimate the percentage of cells in each phase of the cell cycles. This model uses a complex repetitive calculation to produced approximations to the actual histogram, fitting $G_0/G_1$ and $G_2/M$ populations with single Gaussian curves.

### Results

*BM cell viability*

When assayed 7 days after adding 3.5 $\mu g\ ml^{-1}$ CPT, 10 $\mu g\ ml^{-1}$ VP-16 or 10 $\mu g\ ml^{-1}$ doxorubicin to cultures, parental BM cells were nearly all dead (Figure 1). In contrast, the survival of BM cells cultured in the presence of actinomycin D or cycloheximide was maintained at $\geqslant 50\%$ for at least 7 days. These results indicate the loss of BM cell viability induced by CPT, VP-16 or doxorubicin is almost dependent on RNA and protein synthesis. To determine whether Bcl-2 prevents loss of viability induced by these agents, we infected BM cells with a retrovirus bearing genes for both Bcl-2 and G418 resistance or a control virus bearing the gene for G418 resistance alone. Expression of the introduced *bcl*-2 gene was confirmed by immunoprecipitation using anti-human Bcl-2 MAb (Figure 2), and the expression of Bcl-2 protein was maintained for at least 1 month (data not shown). On the other hand, endogenous expression of Bcl-2 in parental BM cells was not detected by this MAb (Figure 2). This MAb does not pick up mouse Bcl-2 protein according to the manufacturer's protocol, but mature polymorphonuclear BM cells are shown to be essentially negative for Bcl-2 expression (Hockenbery *et al.*, 1991). Therefore, we suggest that endogenous expression of Bcl-2 in BM cells may be very less than exogenous expression and not influence our results. In addition, subcloning was unnecessary to achieve BM cells with high levels of *bcl*-2 expression because the gene transfer efficiency was high (positive cells > 90%, determined by an indirect immunofluorescence method using anti-human Bcl-2 MAb), and transfected BM cells showed the almost same level of *bcl*-2 expression (data not shown). About 75% of *bcl*-2-expressing BM cells exposed to chemotherapeutic agents remained viable after 7 days (Figure 1). In contrast, BM cells expressing Neo alone lost viability to the same extent as did the uninfected, parental BM cells. These findings demonstrate that enforced expression of *bcl*-2 increased the resistance of BM cells to chemotherapeutic agents.

*DNA fragmentation*

DNA fragmentation in BM cells exposed to 3.5 $\mu g\ ml^{-1}$ CPT, 10 $\mu g\ ml^{-1}$ VP-16 or 10 $\mu g\ ml^{-1}$ doxorubicin for 48 h at 37°C was determined. Figure 3 shows that the DNA of *bcl*-2-expressing BM cells was intact, whereas that of uninfected BM cells and those expressing Neo alone was broken into nucleosome-sized fragments. These results demonstrate that the expression of *bcl*-2 gene was nearly sufficient to maintain the integrity of DNA in BM cells treated with chemotherapeutic agents.

### *Ultrastructural appearance*

Figure 4 shows that parental BM cells treated with VP-16 lost viability and frequently displayed typical apoptotic morphology including chromatin condensation, while the viability of *bcl*-2-expressing BM cells remained high. However, about 20% of *bcl*-2-expressing BM cells were also dead; the mechanism of cell death was apoptosis (data not shown).

### *Cell cycle*

We examined the changes in the intensity of fluorescence of DNA using flow cytometry. As shown in Figure 5, VP-16 treatment of parental BM cells resulted in a decrease in the percentage of cells in $G_0/G_1$ phase and an increase in percentage of cells in S and $G_2/M$ phases, compared with the corresponding percentage for the control. Moreover, treatment of parental BM cells with VP-16 resulted in the accumulation of a discrete subpopulation of signals under the $G_0/G_1$ cell cycle region ($A_0$ peak). In contrast, *bcl*-2 inhibited the appearance of this sub-$G_0/G_1$ peak in the DNA histograms.

**Figure 1** Survival kinetics of BM cells exposed to CPT (3.5 μg ml$^{-1}$, **a**), VP-16 (10 μg ml$^{-1}$, **b**) or doxorubicin (10 μg ml$^{-1}$, **c**). BM cells were seeded at a density of $10^5$ cells ml$^{-1}$ and incubated at 37°C. Viability was determined at each time point by trypan blue exclusion. Values represent the mean ± s.d. of results of three experiments. ○, ◆ or +, parental BM cells in the absence or in the presence of actinomycin D (80 ng ml$^{-1}$) or cycloheximide (0.7 μg ml$^{-1}$); Δ, *neo*-expressing BM cells; □, *bcl*-2-expressing BM cells.

**Figure 2** Immunoprecipitation with anti-human Bcl-2 MAb. Parental (lane 1), *bcl*-2-infected (lane 2) and *neo*-infected (lane 3) BM cells were labelled with [$^{35}$S]L-methionine, lysed and immunoprecipitated with anti-human Bcl-2 MAb. The immunoprecipitates were analysed on a 12% sodium dodecyl sulphate-polyacrylamide gel.

**Figure 3** Induction of DNA fragmentation by chemotherapeutic agents. DNA fragmentation was assessed for parental (lane 2, 5, or 8), *neo*-(lane 3, 6 or 9) and *bcl*-2-expressing (lane 4, 7 or 10) BM cells treated with CPT (3.5 μg ml$^{-1}$; lanes 2–4), VP-16 (10 μg ml$^{-1}$; lanes 5–7) or doxorubicin (10 μg ml$^{-1}$; lanes 8–10) for 48 h. Fragmented DNA was electrophoresed in a 2.0% agarose gel containing 0.5 μg ml$^{-1}$ ethidium bromide. Molecular weight standards of multiples of 123-bp DNA ladder (Gibco BRL, Tokyo) are shown in lane 1.

**Figure 4** Ultrastructural appearance of parental **a**, and *bcl*-2-expressing **b**, BM cells treated with 10 μg ml$^{-1}$ VP-16 for 48 h ( × 5,600). Arrow indicates condensed chromatin.

**Figure 5** Flow cytometric analysis of BM cells treated without (control, **a**) or with 10 μg ml$^{-1}$ VP-16, **b** or **c**, for 48 h. Parental **a** or **b** and *bcl*-2-expressing **c**, BM cells were subsequently fixed and stained with propidium iodide prior to DNA histogram analysis. In each case cell number (ordinate) was plotted against relative fluorescence intensity (abscissa). The percentages of cells in each phase of the cell cycle at $A_0$ (a subpopulation of signals under the $G_0/G_1$ cell cycle region): $G_0/G_1$:S:$G_2$/M: **a**, 0:48:47:5; **b**, 11:14:53:22; **c**, 0:59:30:11.

### Discussion

Nuclear enzymes, in particular DNA topoisomerases, function in cellular proliferation and differentiation by inducing changes in the topology of DNA, enabling DNA synthesis, recombination and transcription (Wang, 1985; Liu, 1989). During topological transformation reactions, topoisomerase I generates transient single-strand DNA breaks and relieves torsional stress by untwisting the DNA helix, while topoisomerase II introduces transient double-strand DNA breaks, resolves DNA molecule tangles, and untwists the DNA helix. CPT has been shown to inhibit topoisomerase I through the formation of stable topoisomerase I-DNA cleavable complexes. Also, anti-tumour agents such as epipodophyllotoxin (VP-16) and doxorubicin (Adriamycin) have been thought to exert effects via interaction with topoisomerase II. However, the exact mechanism by which interaction of these agents with the topoisomerase I or II-DNA cleavable complex leads to cell death is unclear. In this study, we show that topoisomerase inhibitors induce apoptosis in BM cells in the presence of new RNA and protein synthesis. This active process, apoptosis of BM cells, may be the main mechanism of drug-induced myelosuppression, since the nadir of peripheral neutrophils occurred usually on days 7-10 post treatment of VP-16 and doxorubicin (Wakui et al., 1986; Bronchud et al., 1989).

In this study, in addition, VP-16 induced a marked reduction in $G_0/G_1$ phase cells and a large increase in the number of S and $G_2$/M cells. These findings suggest two possibilities: that BM cell death occurs directly out of $G_0/G_1$ phase or, alternatively, that cells continue to cycle in the presence of VP-16 and die at a later stage in the cell cycle. Moreover, the accumulation of $A_0$ peak was shown; this peak has been shown to indicate the presence of apoptotic cells (Telford et al., 1991; Walker et al., 1991; Del Bino et al., 1992). In general, topoisomerase II inhibitors are thought to kill proliferating cells by inhibiting topoisomerase II and preventing the cells from either completing S phase or undergoing chromosome segregation at mitosis since these are two cellular processes that have an absolute requirement for the enzyme (Walker *et al.*, 1991). This cytotoxicity has been shown to correlate with drug-induced DNA cleavage and to increase when the drugs are administered during these phases of the cell cycle (Long & Stringfellow, 1988). In contrast, Estey *et al.* (1987) indicate that cells are hypersensitive to topoisomerase II inhibitors in mitosis but that the hypersensitivity does not correlate with cytotoxicity. Furthermore, Kaufmann (1988) has demonstrated that there is no direct correlation between the ability of topoisomerase II inhibitors to cause DNA strand breaks and their ability to induce cell death via apoptosis. Topoisomerase II inhibitor-induced strand breaks are rapidly resealed after removal of the drugs, while this resealing does not inhibit the onset of apoptosis. Taken together, further experiments are necessary to determine during which phase apoptosis in BM cells is occurring.

The proto-oncogene *bcl*-2 was discovered as a result of its translocation to the immunoglobulin heavy-chain locus in most cases of human follicular centre B-cell lymphoma (Bakhshi *et al.*, 1985; Cleary & Sklar, 1985; Vaux *et al.*, 1988). This t(14;18) chromosomal translocation spares the coding region of the *bcl*-2 gene but appears to deregulate its expression. The *bcl*-2 gene encodes a cytoplasmic protein (Tsujimoto *et al.*, 1987; Chen-Levy *et al.*, 1989) that appears to be associated with the inner membrane of the mitochondria (Hockenbery *et al.*, 1990). Insight into the biological function of Bcl-2 came with the discovery that enforced *bcl*-2 expression delays the death of certain haematopoietic cell lines deprived of growth factor (Cleary & Sklar, 1985). However, the mechanism by which the *bcl*-2 gene regulates cell viability remains unclear, since the predicted amino acid sequence of the protein it codes for bears no significant homology to other known proteins and no biochemical activity has yet been ascribed to it (Miyashita & Reed, 1992). More recently, Hockenbery *et al.* (1993) demonstrate that Bcl-2 functions in an antioxidant pathway to prevent apoptosis. Moreover, transgenic mice expressing a *bcl*-2 gene subjected to an immunoglobulin enhancer contain a large excess of B lymphocytes with enhanced survival capacity that may progress into high-grade lymphoma or autoimmune disease (McDonnell *et al.*, 1990; Strasser *et al.*, 1991), and Bcl-2 confers survival advantage on Epstein–Barr virus-infected B cells (Nunez *et al.*, 1990; Henderson *et al.*, 1991). Taken together, it will be a problem to introduce *bcl*-2, an oncogene, into human BM, as it could cause tumours or autoimmune disease.

In this study, we also show that enforced expression of *bcl*-2 gene in BM cells results in increased resistance to apoptosis induced by topoisomerase inhibitors. Our results are essentially in agreement with Miyashita and Reed (1992, 1993), who have recently demonstrated that *bcl*-2 gene transfer increases the relative resistance or murine lymphoid cells and human leukaemia cells to cell death and DNA fragmentation induced by chemotherapeutic drugs such as methotrexate, vincristine (Miyashita & Reed, 1992) and VP-16 (Miyashita & Reed, 1993). The *bcl*-2 gene might therefore interfere with a final common pathway for cell death that can be activated by multiple mechanisms.

In conclusion, our findings suggest the possibility that enforced expression of the *bcl*-2 gene in BM cells using gene transfer techniques may enable rescue from myelosuppression (BM cell death) induced by chemotherapeutic agents such as topoisomerase inhibitors. We are at present attempting to determine whether the *bcl*-2 gene can be used to rescue chemotherapy-induced myelosuppression in an *in vivo* model, without inhibiting the cytotoxic effect of agents on tumour cells.

**Abbreviations**

rhG-CSF, recombinant human granulocyte colony-stimulating factor; CPT, camptothecin; VP-16, etoposide; MAb, monoclonal antibody.

We thank Dr D.L. Vaux for kind gifts of murine fibroblast lines secreting MPZenNeo (bcl-2) or MPZenNeo virus. We also thank Ms M. Nakajima and Ms E. Nishiguchi for technical assistance. This work was supported in part by the Grant-in Aid for Cancer Research (5-6) from the Ministry of Health and Welfare of Japan.

**References**

ANTMAN, K.S., GRIFFIN, J.D., ELIAS, A., SOCINSKI, M.A., RYAN, L., CAMMISTRA, S.A., OETTE, D., WHITLEY, M., FREI III, E. & SCHNIPPER, L.E. (1988). Effect of recombinant human granulocyte–macrophage colony-stimulating factor on chemotherapy-induced myelosuppression. *N. Engl. J. Med.*, **319**, 593–598.

BAKHSHI, A., JENSEN, J.P., GOLDMAN, P.J., WRIGHT, J., MCBRIDE, O.W., EPSTEIN, A.L. & KORSMEYER, S.J. (1985). Cloning the chromosomal breakpoint of t(14:18) human lymphomas: clustering around $J_H$ on chromosome 14 and near a transcription unit on 18. *Cell*, **41**, 899–906.

BRANDT, S.J., PETERS, W.P., ATWATER, S.K., KURTZBERG, J., BOROWITZ, M.J., JONES, R.B., SHPALL, E.J., BAST, R.C., GILBERT, C.J. & OETTE, D.H. (1988). Effect of recombinant human granulocyte–macrophage colony-stimulating factor on hematopoietic reconstitution after high-dose chemotherapy and autologous bone marrow transplantation. *N. Engl. J. Med.*, **318**, 869–876.

BRONCHUD, M.H., HOWELL, A., CROWTHER, D., HOPWOOD, P., SOUZA, L. & DEXTER, T.M. (1989). The use of granulocyte colony-stimulating factor to increase the intensity of treatment with doxorubicin in patients with advanced breast and ovarian cancer. *Br. J. Cancer*, **60**, 121–125.

CHEN-LEVY, Z., NOURSE, J. & CLEARY, M.L. (1989). The *bcl*-2 candidate proto-oncogene product is a 24-kilodalton integral-membrane protein highly expressed in lymphoid cell lines and lymphomas carrying the t(14;18) translocation. *Mol Cell. Biol.*, **9**, 701–710.

CLEARY, M.L. & SKLAR, J. (1985). Nucleotide sequence of a t(14;18) chromosomal breakpoint in follicular lymphoma and demonstration of a breakpoint-cluster region near a transcriptionally active locus on chromosome 18. *Proc. Natl. Acad. Sci. USA*, **82**, 7439–7443.

CLEARY, M.L., SMITH, S.D. & SKLAR, J. (1986). Cloning and structural analysis of cDNAs for bcl-2 and a hybrid bcl-2 immunoglobulin transcript resulting from the t(14;18) translocation. *Cell*, **47**, 19–28.

DEL BINO, G., BRUNO, S., YI, P.N. & DARZYNKIEWICZ, Z. (1992). Apoptotic cell death triggered by camptothecin or teniposide. The cell cycle specificity and effects of ionizing radiation. *Cell Prolif.*, **25**, 537–548.

EASTMAN, A. (1990). Activation of programmed cell death by anticancer agents: cisplatin as a model system. *Cancer Cells*, **2**, 275–280.

ESTEY, E., ADLAKHA, R.C., HITTELMAN, W.N. & ZWELLING, L.A. (1987). Cell cycle stage dependent variations in drug-induced topoisomerase II mediated DNA cleavage and cytotoxicity. *Biochemistry*, **26**, 4338–4344.

GLISSON, B.S. & ROSS, W.E. (1987). DNA topoisomerase II: a primer on the enzyme and its unique role as a multidrug target in cancer chemotherapy. *Pharmacol. Ther.*, **32**, 89–106.

HARIHARAN, I.K., ADAMS, J.M. & CORY, S. (1988). Bcr-abl oncogene renders myeloid cell line factor independent: potential autocrine mechanism in chronic myeloid leukemia. *Oncogene Res.*, **3**, 387–399.

HENDERSON, S., ROWE, M., GREGORY, C., CROOM-CARTER, D., WANG, F., LONGNECKER, R., KIEFF, E. & RICKINSON, A. (1991). Induction of bcl-2 expression by Epstein–Barr virus latent membrane protein 1 protects infected B cells from programmed cell death. *Cell*, **65**, 1107–1115.

HOCKENBERY, D., NUNEZ, G., MILLIMAN, C., SCHREIBER, R.D. & KORSMEYER, S.J. (1990). Bcl-2 is an inner mitochondrial membrane protein that blocks programmed cell death. *Nature*, **348**, 334–336.

HOCKENBERY, D.M., ZUTTER, M., HICKEY, W., NAHM, M. & KORSMEYER, S.J. (1991). BCL2 protein is topographically restricted in tissues characterized by apoptotic cell death. *Proc. Natl. Acad. Sci. USA*, **88**, 6961–6965.

HOCKENBERY, D.M., OLTVAI, Z.N., YIN, X.-M., MILLIMAN, C.L. & KORSMEYER, S.J. (1993). Bcl-2 functions in an antioxidant pathway to prevent apoptosis. *Cell*, **75**, 241–251.

ISHIDA, Y., AGATA, Y., SHIBAHARA, K. & HONJO, T. (1992). Induced expression of PD-1, a novel member of the immunoglobulin gene superfamily, upon programmed cell death. *EMBO J.*, **11**, 3887–3895.

JAXEL, C., TADOU, G., PORTEMER, C., MIRAMBEAU, G., PANIJEL, J. & DUGUET, M. (1988). Topoisomerase inhibitors induce irreversible fragmentation of replicated DNA in concanavalin A stimulated splenocytes. *Biochemistry*, **27**, 95–99.

KAUFMANN, S.H. (1989). Induction of endonucleolytic DNA cleavage in human acute myelogenous leukemia cells by etoposide, camptothecin, and other cytotoxic anti-cancer drugs: a cautionary note. *Cancer Res.*, **49**, 5870–5878.

KOHN, K.W., POMMIER, Y., KERRIGAN, D., MARKOVITS, J. & COVEY, J.M. (1987). Topoisomerase II as a target of anticancer drug action in mammalian cells. *Natl. Cancer Inst. Monogr.*, **4**, 61–71.

KONDO, S., MIYATAKE, S., IWASKAI, K., ODA, Y., KIKUCHI, H., ZU, Y., SHAMOTO, M. & NAMBA, Y. (1992). Human glioma-specific antigens detected by monoclonal antibodies. *Neurosurgery*, **30**, 506–511.

LIU, L.F. (1989). DNA topoisomerase poisons as antitumor drugs. *Annu. Rev. Biochem.*, **58**, 351–375.

LONG, B.H. & STRINGFELLOW, D.A. (1988). Inhibitors of topoisomerase II: structure–activity relationships and mechanism of action of podophyllin congeners. *Adv. Enzyme Regul.*, **27**, 223–256.

LOTEM, J. & SACHS, L. (1992). Hematopoietic cytokines inhibit apoptosis induced by transforming growth factor $\beta$1 and cancer chemotherapy compounds in myeloid leukemic cells. *Blood*, **80**, 1750–1757.

McDONNELL, T.J., NUNEZ, G., PLATT, F.M., HOCKENBERY, D., LONDON, L., McKEARN, J.P. & KORSMEYER, S.J. (1990). Deregulated bcl-2-immunoglobulin transgene expands a resting but responsive immunoglobulin M and D-expressing B-cell population. *Mol. Cell. Biol.*, **10**, 1901–1907.

MANN, R., MULLIGAN, R.C. & BALTIMORE, D. (1983). Construction of a retrovirus packaging mutant and its use to produce helper-free defective retrovirus. *Cell*, **33**, 153–157.

MARTIN, S.J. & COTTER, T.G. (1991). Disruption of microtubules induces an endogenous suicide pathway in human leukemia HL-60 cells. *Cell Tissue Kinetics*, **23**, 545–559.

MARTIN, S.J., LENNON, S.V., BONHAM, A.M. & COTTER, T.G. (1990). Induction of apoptosis (programmed cell death) in human leukemic HL-60 cells by inhibition of RNA and protein synthesis. *J. Immunol.*, **145**, 1859–1867.

MIYASHITA, T. & REED, J.C. (1992). bcl-2 gene transfer increases relative resistance of S49.1 and WEHI7.2 lymphoid cells to cell death and DNA fragmentation induced by glucocorticoids and multiple chemotherapeutic drugs. *Cancer Res.*, **52**, 5407–5411.

MIYASHITA, T. & REED, J.C. (1993). Bcl-2 oncoprotein blocks chemotherapy-induced apoptosis in a human leukemia cell line. *Blood*, **81**, 151–157.

NUNEZ, G., LONDON, L., HOCKENBERY, D., ALEXANDER, M., McKEARN, J.P. & KORSMEYER, S.J. (1990). Deregulated bcl-2 gene expression selectively prolongs survival of growth factor-deprived hempoietic cell lines. *J. Immunol.*, **144**, 3602–3610.

ONISHI, Y., AZUMA, Y., SATO, Y., MIZUNO, Y., TADAKUMA, T. & KIZAKI, H. (1993). Topoisomerase inhibitors induce apoptosis in thymocytes. *Biochim. Biophys. Acta*, **1175**, 147–154.

SORENSON, C.M., BARRY, M.A. & EASTMAN, A. (1990). Analysis of events associated with cell cycle arrest in $G_2$ phase and cell death induced by cisplatin. *J. Natl Cancer Inst.*, **82**, 749–755.

STRASSER, A., WHITTINGHAM, S., VAUX, D.L., BATH, M.L., ADAMS, J.M., CORY, S. & HARRIS, A.W. (1991). Enforced BCL2 expression in B-lymphoid cells prolongs antibody responses and elicits autoimmune disease. *Proc. Natl. Acad. Sci. USA*, **88**, 8661–8665.

STRASSMAN, G., COLE, M.D. & NEWMAN, W. (1988). Regulation of colony-stimulating factor-dependent macrophage precursor proliferation by type $\beta$ transforming growth factor. *J. Immunol.*, **140**, 2645–2651.

TELFORD, W.G., KING, L.E. & FRAKER, P.J. (1991). Evaluation of glucocorticoid-induced DNA fragmentation in mouse thymocytes by flow cytometry. *Cell Prolif.*, **24**, 447–459.

THAKKAR, N.S. & POTTEN, C.S. (1992). Abrogation of adriamycin toxicity in vivo by cycloheximide. *Biochem. Pharmacol.*, **43**, 1683–1691.

TSUJIMOTO, Y. (1989). Stress-resistance conferred by high level of bcl-2$\alpha$ protein in human B lymphoblastoid cell. *Oncogene*, **4**, 1331–1336.

TSUJIMOTO, Y., COSSMAN, J., JAFFE, E. & CROCE, C.M. (1984). Cloning of the breakpoint of neoplastic B cells with the t(14;18) chromosome translocation. *Science*, **226**, 1097–1099.

TSUJIMOTO, Y., IKEGAKI, N. & CROCE, C.M. (1987). Characterization of the protein product of bcl-2, the gene involved in human follicular lymphoma. *Oncogene*, **2**, 3–7.

VAUX, D.L., CORY, S. & ADAMS, J.M. (1988). bcl-2 gene promotes haemopoietic cell survival and cooperates with c-myc to immortalize pre-B cells. *Nature*, **335**, 440–442.

WAKUI, A., YOKOYAMA, M., TAKAHASHI, H., YOSHIDA, Y., SAKATA, Y., SATO, S., KANO, A., KAWAMOTO, K., HASHIMOTO, S., KONNO, K., KOINUMARU, S., NAKAI, Y., MASAMUNE, O., INOUE, Y., MIURA, A., AKIHAMA, T., SUZUKI, K., NUMASAWA, K., ENDO, S., WATANABE, I., SUZUKI, M., SAITO, T. & NAKAO, I. (1986). A phase I study of VP-16-213 (VP, etoposide) by a single and 5-day intravenous administration. *Jpn. J. Cancer Chemother.*, **13**, 319–329.

WALKER, P.R., SMITH, C., YOUDALE, T., LEBLANC, J., WHITFIELD, J.F. & SIKORSKA, M. (1991). Topoisomerase II-reactive chemotherapeutic drugs induce apoptosis in thymocytes. *Cancer Res.*, **51**, 1078–1085.

WANG, J.C. (1985). DNA topoisomerases. *Annu. Rev. Biochem.*, **54**, 665–697.

ZWELLING, L.A. (1989). Topoisomerase II as a target of antileukemia drugs: a review of controversial areas. *Hum. Pathol.*, **3**, 101–112.


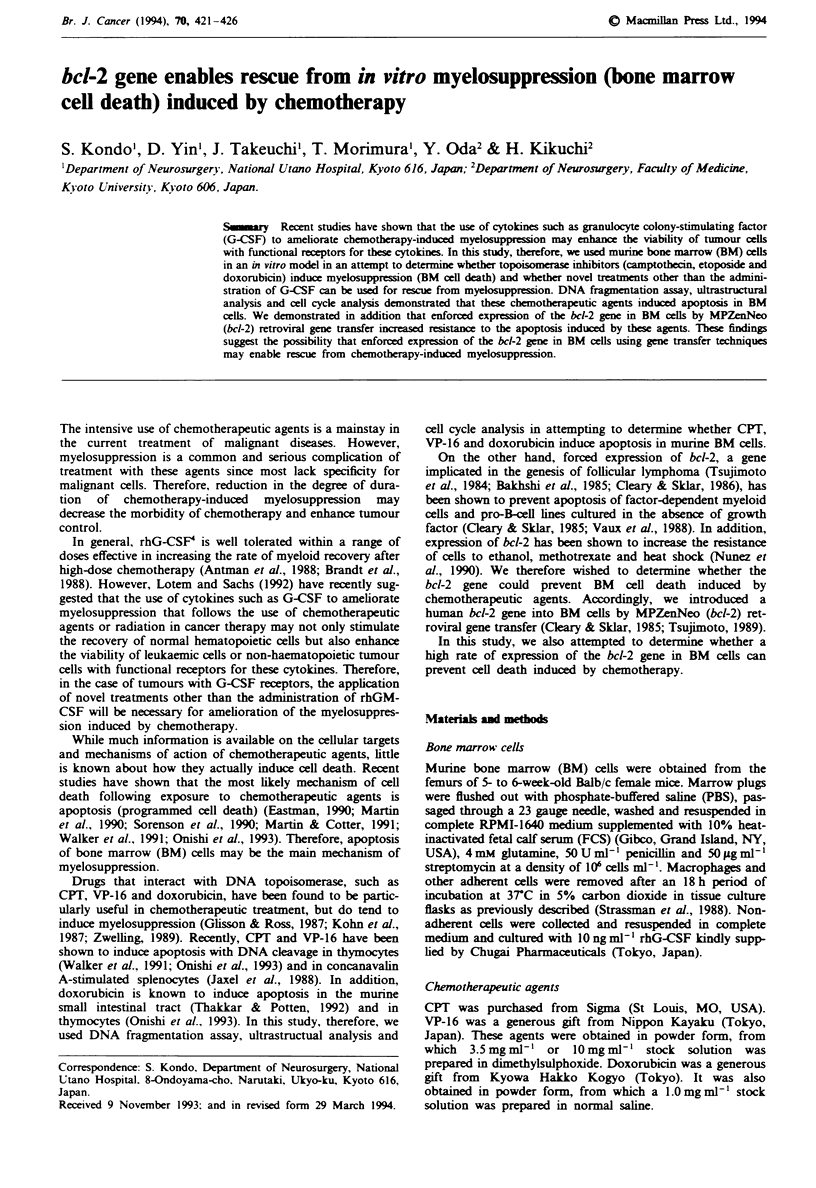

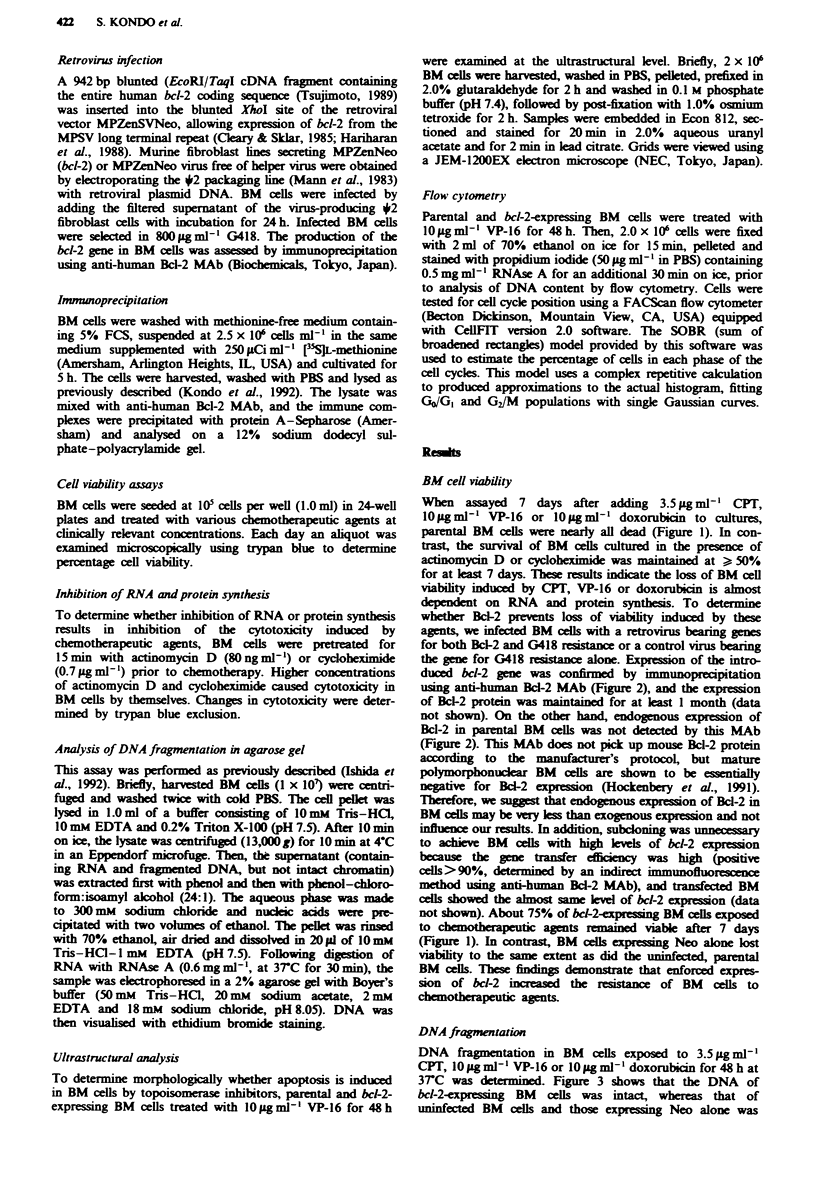

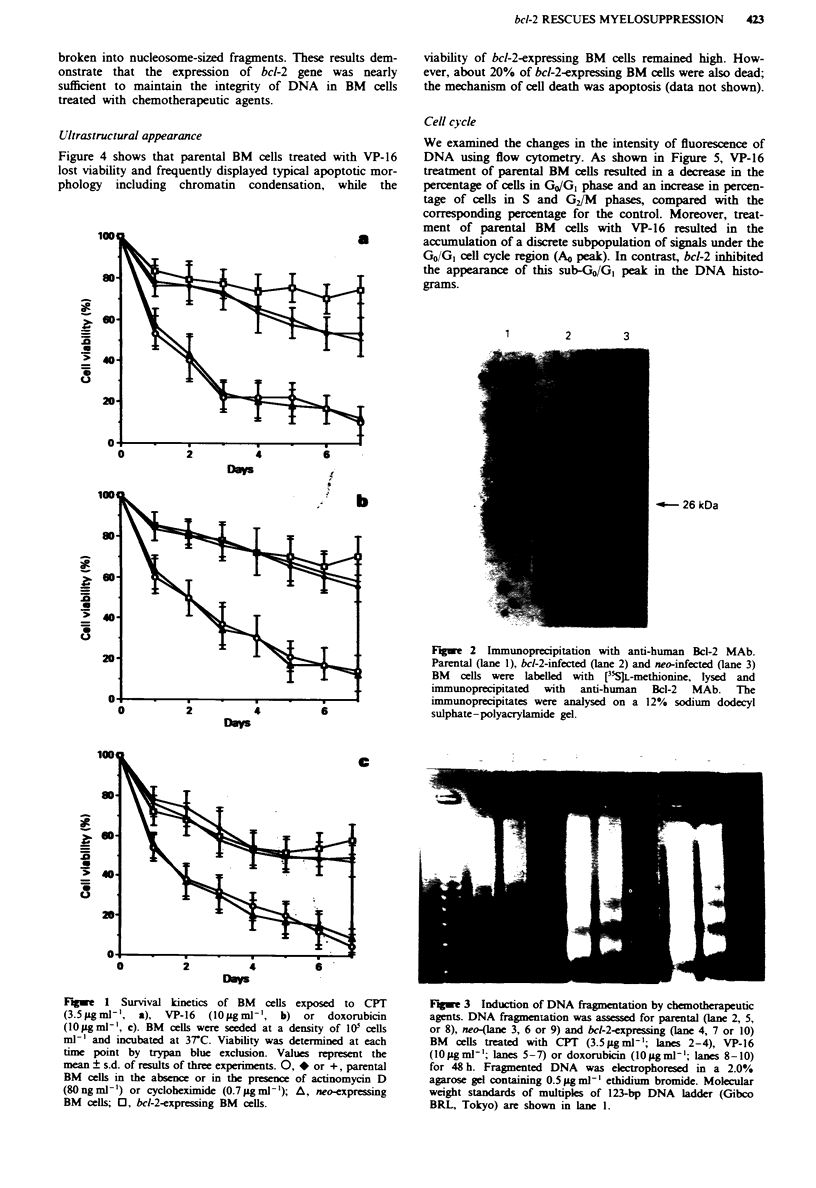

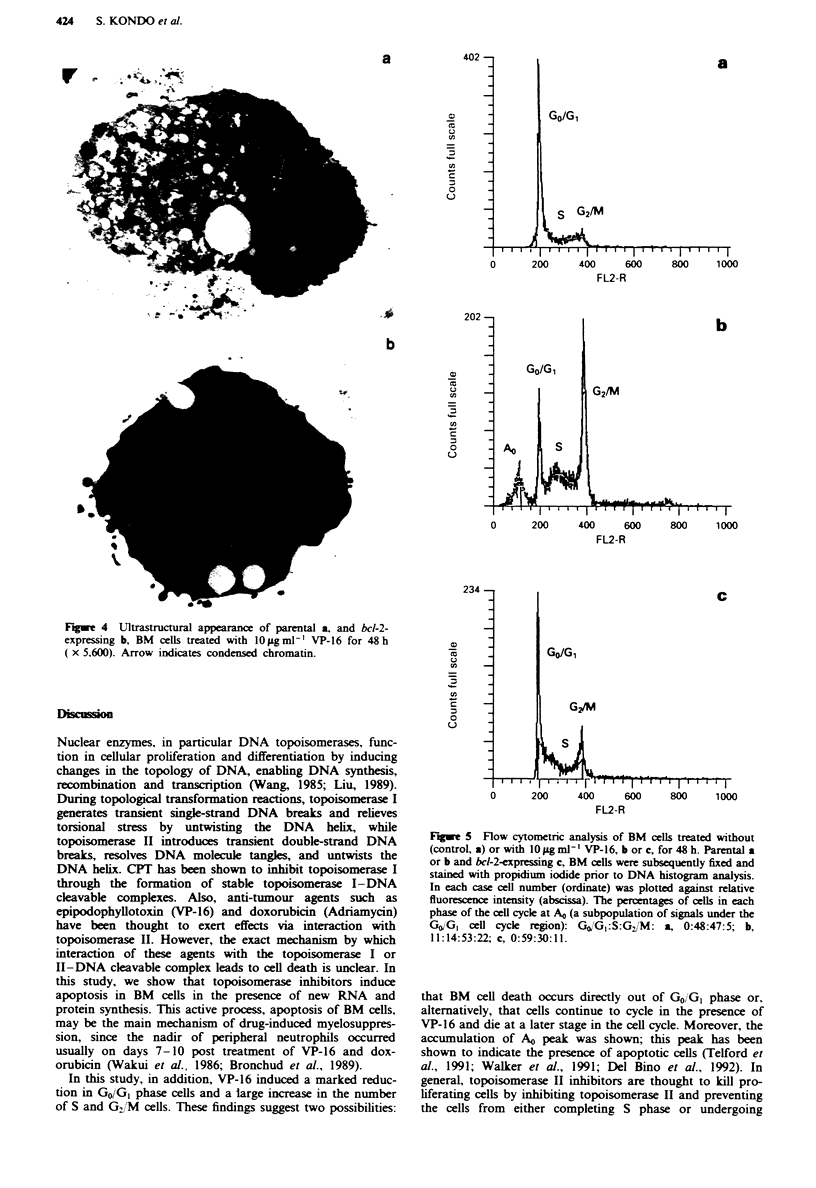

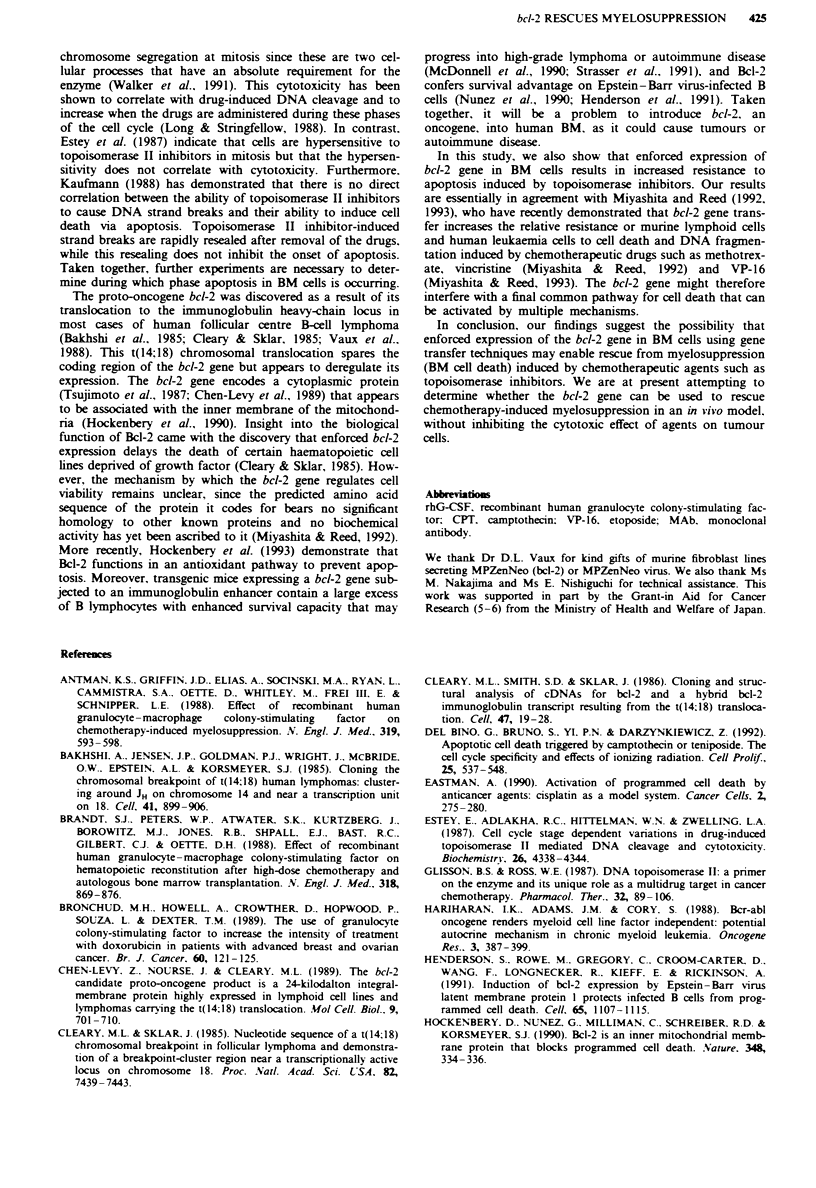

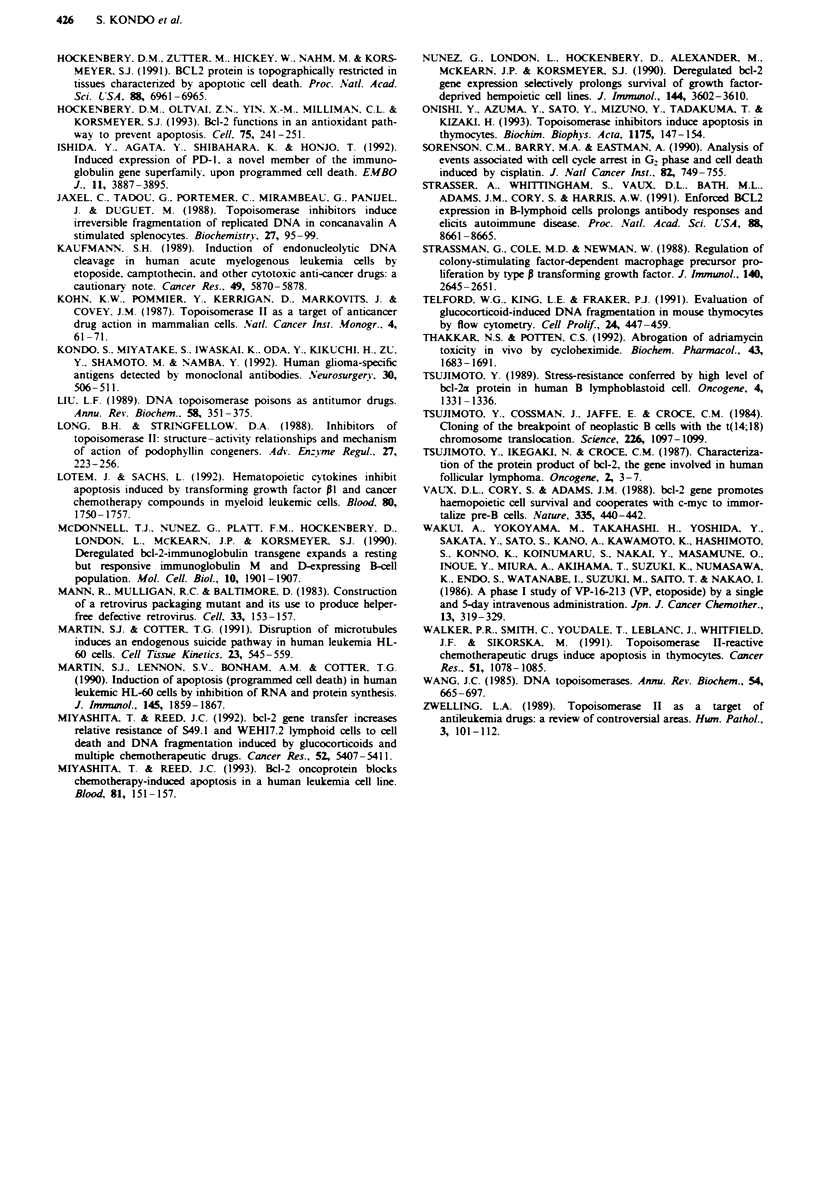


## References

[OCR_00618] Antman K. S., Griffin J. D., Elias A., Socinski M. A., Ryan L., Cannistra S. A., Oette D., Whitley M., Frei E., Schnipper L. E. (1988). Effect of recombinant human granulocyte-macrophage colony-stimulating factor on chemotherapy-induced myelosuppression.. N Engl J Med.

[OCR_00623] Bakhshi A., Jensen J. P., Goldman P., Wright J. J., McBride O. W., Epstein A. L., Korsmeyer S. J. (1985). Cloning the chromosomal breakpoint of t(14;18) human lymphomas: clustering around JH on chromosome 14 and near a transcriptional unit on 18.. Cell.

[OCR_00630] Brandt S. J., Peters W. P., Atwater S. K., Kurtzberg J., Borowitz M. J., Jones R. B., Shpall E. J., Bast R. C., Gilbert C. J., Oette D. H. (1988). Effect of recombinant human granulocyte-macrophage colony-stimulating factor on hematopoietic reconstitution after high-dose chemotherapy and autologous bone marrow transplantation.. N Engl J Med.

[OCR_00639] Bronchud M. H., Howell A., Crowther D., Hopwood P., Souza L., Dexter T. M. (1989). The use of granulocyte colony-stimulating factor to increase the intensity of treatment with doxorubicin in patients with advanced breast and ovarian cancer.. Br J Cancer.

[OCR_00646] Chen-Levy Z., Nourse J., Cleary M. L. (1989). The bcl-2 candidate proto-oncogene product is a 24-kilodalton integral-membrane protein highly expressed in lymphoid cell lines and lymphomas carrying the t(14;18) translocation.. Mol Cell Biol.

[OCR_00655] Cleary M. L., Sklar J. (1985). Nucleotide sequence of a t(14;18) chromosomal breakpoint in follicular lymphoma and demonstration of a breakpoint-cluster region near a transcriptionally active locus on chromosome 18.. Proc Natl Acad Sci U S A.

[OCR_00660] Cleary M. L., Smith S. D., Sklar J. (1986). Cloning and structural analysis of cDNAs for bcl-2 and a hybrid bcl-2/immunoglobulin transcript resulting from the t(14;18) translocation.. Cell.

[OCR_00666] Del Bino G., Bruno S., Yi P. N., Darzynkiewicz Z. (1992). Apoptotic cell death triggered by camptothecin or teniposide. The cell cycle specificity and effects of ionizing radiation.. Cell Prolif.

[OCR_00672] Eastman A. (1990). Activation of programmed cell death by anticancer agents: cisplatin as a model system.. Cancer Cells.

[OCR_00677] Estey E., Adlakha R. C., Hittelman W. N., Zwelling L. A. (1987). Cell cycle stage dependent variations in drug-induced topoisomerase II mediated DNA cleavage and cytotoxicity.. Biochemistry.

[OCR_00685] Glisson B. S., Ross W. E. (1987). DNA topoisomerase II: a primer on the enzyme and its unique role as a multidrug target in cancer chemotherapy.. Pharmacol Ther.

[OCR_00688] Hariharan I. K., Adams J. M., Cory S. (1988). bcr-abl oncogene renders myeloid cell line factor independent: potential autocrine mechanism in chronic myeloid leukemia.. Oncogene Res.

[OCR_00694] Henderson S., Rowe M., Gregory C., Croom-Carter D., Wang F., Longnecker R., Kieff E., Rickinson A. (1991). Induction of bcl-2 expression by Epstein-Barr virus latent membrane protein 1 protects infected B cells from programmed cell death.. Cell.

[OCR_00715] Hockenbery D. M., Oltvai Z. N., Yin X. M., Milliman C. L., Korsmeyer S. J. (1993). Bcl-2 functions in an antioxidant pathway to prevent apoptosis.. Cell.

[OCR_00709] Hockenbery D. M., Zutter M., Hickey W., Nahm M., Korsmeyer S. J. (1991). BCL2 protein is topographically restricted in tissues characterized by apoptotic cell death.. Proc Natl Acad Sci U S A.

[OCR_00701] Hockenbery D., Nuñez G., Milliman C., Schreiber R. D., Korsmeyer S. J. (1990). Bcl-2 is an inner mitochondrial membrane protein that blocks programmed cell death.. Nature.

[OCR_00720] Ishida Y., Agata Y., Shibahara K., Honjo T. (1992). Induced expression of PD-1, a novel member of the immunoglobulin gene superfamily, upon programmed cell death.. EMBO J.

[OCR_00728] Jaxel C., Taudou G., Portemer C., Mirambeau G., Panijel J., Duguet M. (1988). Topoisomerase inhibitors induce irreversible fragmentation of replicated DNA in concanavalin A stimulated splenocytes.. Biochemistry.

[OCR_00734] Kaufmann S. H. (1989). Induction of endonucleolytic DNA cleavage in human acute myelogenous leukemia cells by etoposide, camptothecin, and other cytotoxic anticancer drugs: a cautionary note.. Cancer Res.

[OCR_00738] Kohn K. W., Pommier Y., Kerrigan D., Markovits J., Covey J. M. (1987). Topoisomerase II as a target of anticancer drug action in mammalian cells.. NCI Monogr.

[OCR_00744] Kondo S., Miyatake S., Iwasaki K., Oda Y., Kikuchi H., Zu Y., Shamoto M., Namba Y. (1992). Human glioma-specific antigens detected by monoclonal antibodies.. Neurosurgery.

[OCR_00750] Liu L. F. (1989). DNA topoisomerase poisons as antitumor drugs.. Annu Rev Biochem.

[OCR_00756] Long B. H., Stringfellow D. A. (1988). Inhibitors of topoisomerase II: structure-activity relationships and mechanism of action of podophyllin congeners.. Adv Enzyme Regul.

[OCR_00760] Lotem J., Sachs L. (1992). Hematopoietic cytokines inhibit apoptosis induced by transforming growth factor beta 1 and cancer chemotherapy compounds in myeloid leukemic cells.. Blood.

[OCR_00773] Mann R., Mulligan R. C., Baltimore D. (1983). Construction of a retrovirus packaging mutant and its use to produce helper-free defective retrovirus.. Cell.

[OCR_00778] Martin S. J., Cotter T. G. (1990). Disruption of microtubules induces an endogenous suicide pathway in human leukaemia HL-60 cells.. Cell Tissue Kinet.

[OCR_00785] Martin S. J., Lennon S. V., Bonham A. M., Cotter T. G. (1990). Induction of apoptosis (programmed cell death) in human leukemic HL-60 cells by inhibition of RNA or protein synthesis.. J Immunol.

[OCR_00766] McDonnell T. J., Nunez G., Platt F. M., Hockenberry D., London L., McKearn J. P., Korsmeyer S. J. (1990). Deregulated Bcl-2-immunoglobulin transgene expands a resting but responsive immunoglobulin M and D-expressing B-cell population.. Mol Cell Biol.

[OCR_00795] Miyashita T., Reed J. C. (1993). Bcl-2 oncoprotein blocks chemotherapy-induced apoptosis in a human leukemia cell line.. Blood.

[OCR_00791] Miyashita T., Reed J. C. (1992). bcl-2 gene transfer increases relative resistance of S49.1 and WEHI7.2 lymphoid cells to cell death and DNA fragmentation induced by glucocorticoids and multiple chemotherapeutic drugs.. Cancer Res.

[OCR_00800] Nuñez G., London L., Hockenbery D., Alexander M., McKearn J. P., Korsmeyer S. J. (1990). Deregulated Bcl-2 gene expression selectively prolongs survival of growth factor-deprived hemopoietic cell lines.. J Immunol.

[OCR_00808] Onishi Y., Azuma Y., Sato Y., Mizuno Y., Tadakuma T., Kizaki H. (1993). Topoisomerase inhibitors induce apoptosis in thymocytes.. Biochim Biophys Acta.

[OCR_00811] Sorenson C. M., Barry M. A., Eastman A. (1990). Analysis of events associated with cell cycle arrest at G2 phase and cell death induced by cisplatin.. J Natl Cancer Inst.

[OCR_00816] Strasser A., Whittingham S., Vaux D. L., Bath M. L., Adams J. M., Cory S., Harris A. W. (1991). Enforced BCL2 expression in B-lymphoid cells prolongs antibody responses and elicits autoimmune disease.. Proc Natl Acad Sci U S A.

[OCR_00823] Strassmann G., Cole M. D., Newman W. (1988). Regulation of colony-stimulating factor 1-dependent macrophage precursor proliferation by type beta transforming growth factor.. J Immunol.

[OCR_00829] Telford W. G., King L. E., Fraker P. J. (1991). Evaluation of glucocorticoid-induced DNA fragmentation in mouse thymocytes by flow cytometry.. Cell Prolif.

[OCR_00834] Thakkar N. S., Potten C. S. (1992). Abrogation of adriamycin toxicity in vivo by cycloheximide.. Biochem Pharmacol.

[OCR_00844] Tsujimoto Y., Finger L. R., Yunis J., Nowell P. C., Croce C. M. (1984). Cloning of the chromosome breakpoint of neoplastic B cells with the t(14;18) chromosome translocation.. Science.

[OCR_00849] Tsujimoto Y., Ikegaki N., Croce C. M. (1987). Characterization of the protein product of bcl-2, the gene involved in human follicular lymphoma.. Oncogene.

[OCR_00839] Tsujimoto Y. (1989). Stress-resistance conferred by high level of bcl-2 alpha protein in human B lymphoblastoid cell.. Oncogene.

[OCR_00856] Vaux D. L., Cory S., Adams J. M. (1988). Bcl-2 gene promotes haemopoietic cell survival and cooperates with c-myc to immortalize pre-B cells.. Nature.

[OCR_00869] Walker P. R., Smith C., Youdale T., Leblanc J., Whitfield J. F., Sikorska M. (1991). Topoisomerase II-reactive chemotherapeutic drugs induce apoptosis in thymocytes.. Cancer Res.

[OCR_00879] Zwelling L. A. (1989). Topoisomerase II as a target of antileukemia drugs: a review of controversial areas.. Hematol Pathol.

